# Modeling gene-regulatory networks to describe cell fate transitions and predict master regulators

**DOI:** 10.1038/s41540-018-0066-z

**Published:** 2018-08-02

**Authors:** Pierre-Etienne Cholley, Julien Moehlin, Alexia Rohmer, Vincent Zilliox, Samuel Nicaise, Hinrich Gronemeyer, Marco Antonio Mendoza-Parra

**Affiliations:** 1 Equipe Labellisée Ligue Contre le Cancer, Department of Functional Genomics and Cancer, Institut de Génétique et de Biologie Moléculaire et Cellulaire (IGBMC), Centre National de la Recherche Scientifique UMR 7104, Institut National de la Santé et de la Recherche Médicale U964, University of Strasbourg, Illkirch, France; 2 Present Address: Computational Systems Biology Infrastructure, Chalmers University of Technology, Kemivägen 10, 41296 Gothenburg, Sweden; 3 Present Address: UMR 8030 Génomique Métabolique, Genoscope, Institut François Jacob, CEA, CNRS, University of Evry-val-d’Essonne, University Paris-Saclay, 91057 Évry, France

## Abstract

Complex organisms originate from and are maintained by the information encoded in the genome. A major challenge of systems biology is to develop algorithms that describe the dynamic regulation of genome functions from large omics datasets. Here, we describe TETRAMER, which reconstructs gene-regulatory networks from temporal transcriptome data during cell fate transitions to predict “master” regulators by simulating cascades of temporal transcription-regulatory events.

While previous efforts described cell fate transitions by the reconstruction of transcription factor (TFs)-driven gene-regulatory networks (GRNs) from the analysis of publicly available data (e.g., microarray transcriptomes;^1,2^ Cap Analysis of Gene Expression (CAGE) data;^3^ enriched TF-DNA binding motif analysis^4,5^), none of them considers the inherent temporal dimension of this process. Also, neither provided a modular strategy for reconstructing specific GRNs by incorporating novel information as it becomes available in public databases.

Here, we present TETRAMER (TEmporal TRAnscription regulation ModellER); a Cytoscape App that (i) reconstructs cell fate transition-specific GRNs by integrating user-provided temporal transcriptomes with a variety of pre-established TF-target gene (TG) relationships issued from different types of public information; (ii) predicts master regulator TFs by modeling temporal transcriptional regulation propagation; and (iii) reveals the temporal transcription-regulatory relationships between the TFs participating in cell fate transition (Fig. 1). TETRAMER generates a GRN that includes the temporal evolution of global transcription by using information derived from three sources: GRNs constructed from a plethora of transcriptomes (CellNet^1^), the genome-wide mapping of human promoters and enhancers in multiple cell types/tissues by CAGE of the FANTOM5 consortium (regulatory circuits^3^), and the systematic analysis of ChIP-seq information in the NGS-QC database^6^ (http://ngs-qc.org) **(**Fig.1a**)**.

**Fig. 1:**
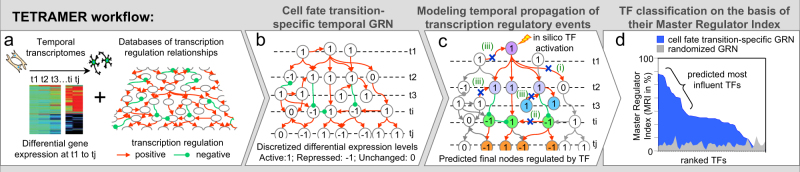
TETRAMER workflow to reconstruct TF regulatory networks by the integrating publicly available GRN information into temporal transcriptomes. **a** TETRAMER reconstructs first a temporal GRN for a cell fate transition by integrating publicly available GRN sources in the temporal transcriptomes established for this transition. **b** Then the temporal propagation of the flux of transcription regulatory information is simulated across the entire GRN, thus establishing a comprehensive connectivity map between all nodes, which represent essentially TFs. For computation, the transcriptional state of each node is discretized (0, 1, -1), as shown. **c** Propagation of the transcription regulatory information applies three logical rules: (i) any connectivity to unresponsive nodes is eliminated, as the signal propagation is terminated; (ii) the flux of information should be coherent between the type of transcription regulation (positive or negative) and the discretized expression level of the interconnected nodes; (iii) the directionality of the transcriptional regulation should comply with the temporal signal flux. Nodes/edges that do not comply with these rules are excluded from the GRN map, as they are not considered specific for the cell fate transition event. Furthermore, nodes/edges downstream of the excluded events are neither considered (herein depicted in gray). **d** Within the reconstituted GRN all nodes are ranked by their master regulator index (MRI), corresponding to the fraction of nodes that are regulated by a given TF upon its activation and signal propagation. The relevance of this ranking is challenged by performing the same procedure in a GRN with randomized connectivities. Thus, TETRAMER identifies master regulator TFs among several thousand differentially expressed genes during cell fate transitions

While informative, the large size of the reconstructed networks (several thousands of nodes and edges) restricts visual tracing of the temporal evolution of the transcription regulatory cascades driving the various types of cell fate transitions. TETRAMER addresses this issue by simulating the propagation of the temporal flux of transcriptional regulation from any TF through the reconstructed network by applying a set of logical rules aiming to avoid the integration of information about TF–TG relationships from heterologous cell/tissue systems, which may be irrelevant for the particular cell fate transition (Fig. 1b, c and Supplementary Fig. 1). Subsequently, TETRAMER evaluates the fraction of regulated genes—relative to a defined population (e.g., defining the terminal state of the cell fate transition)—by any given TF. This fraction; herein referred to as the master regulator index (“MRI”); is further supported by the evaluation of its confidence relative to an MRI issued from the randomization of the GRN connectivity. For this, TETRAMER generates multiple randomly connected GRNs, on the basis of the same nodes and number of edges (up to 100 times), from which a randomized MRI distribution is computed (Supplementary Note). Finally, TETRAMER ranks TFs according to their MRI and depicts the temporally emerging transcription-regulatory landscape in Cytoscape (Fig. 1d and Supplementary Fig. 1).

We have previously used this concept to define a temporal GRN during retinoic acid-induced neuronal differentiation of embryonic stem cells (ESCs).^7^ We reconstructed a GRN (>1900 nodes; >11,600 edges) from six subsequent transcriptomes and queried the temporal evolution of transcription-regulatory cascades emanating from each TF. A subset of ~30 nodes presented MRIs higher than 40% (*p* < 1 × 10^−10^; Supplementary Fig. 2). These nodes not only comprised well-known neurogenic TFs, but also others poorly characterized as major players in neurogenesis. Among them, the early induced factors TAL2, GBX2, DMRT1, or LHX2 were subsequently validated to drive neurogenesis in CRISPR-dCas9 gene activation assays.^7^

To illustrate its versatility, we used TETRAMER to reconstruct dynamic GRNs implicated in iPS cell reprogramming (Supplementary Fig. 3), tumorigenic cell fate transformation (Supplementary Fig. 4), and trans-differentiation of B-cell lymphomas to primary macrophages (Fig. 2). Specifically, re-analysis of temporal transcriptomes generated by Koga and colleagues^8^ for the reprogramming of MEFs to iPSCs identified 21 TFs with MRIs > 25% (*p* < 1 × 10^−10^). Among them, several factors implicated in the maintenance of pluripotency and self-renewal of ESCs (SALL4, SOX2, NANOG, NR0B1, or POU5F1) were shown to be activated at late reprogramming stages (Supplementary Fig. 3). TETRAMER predicted in addition several other factors that were previously reported to be involved in, or enhance reprogramming, like the KLF4-interacting SWI/SNF catalytic subunit SMARCA2/BRM,^9^ the DNA demethylase TET1, which can replace OCT4 in some reprogramming cocktails,^10^ or the PRC2 subunit JARID2.^11,12^

**Fig. 2:**
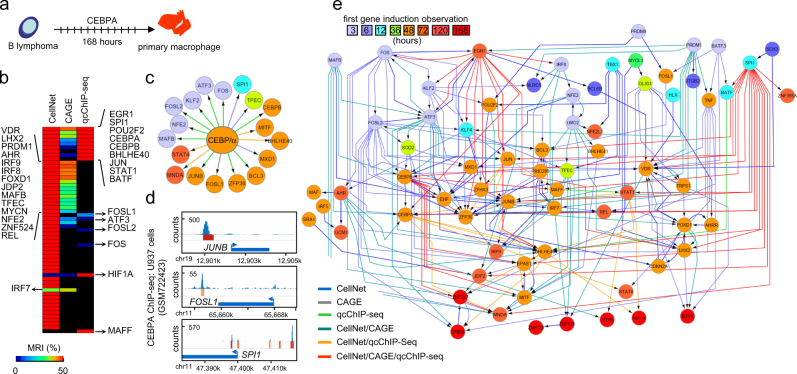
Reconstructing the TF regulatory network involved in B-lymphoma to macrophage trans-differentiation with TETRAMER. **a** TETRAMER was used to model the TF regulatory network implicated in trans-differentiation of B-lymphoma cells to primary macrophages by over-expression of CEBPA.^19^ **b** The information on temporal transcriptional regulation obtained from transcriptomes assessed during the first 168 h after CEBPA over-expression (GSE44700) was combined with the connectivity information obtained from three publicly available GRN sources [CellNet; regulatory circuits established by the FANTOM consortium (regulatorycircuits.org) and systematic reanalysis of all publicly available ChIP-seq datasets (ngs-qc.org)] to predict TFs that acted as master regulators of the trans-differentiation. Heat-maps of TFs identified by integrating each of the three GRN sources were ranked according to their MRI for comparison. **c** C/EBPα cistrome predicted from **(b)**. **d** Verification of three predicted C/EBPα targets in **(c)** by ChIP-sequencing readouts available in the public domain (ngs-qc.org). Note that these three cases were predicted by the various publicly available GRN sources, as indicated by the arrow color-code (displayed in **(e)**). **e** The action of TFs (nodes are color-coded according to gene induction, as indicated at the top) in a GRN generated with TETRAMER, highlighting their temporal regulation during B-lymphoma to macrophage trans-differentiation. The origin of the integrated edges is color-coded to reveal the corresponding connectivity data sources

We previously reported that tumorigenesis of pre-transformed human fibroblasts is induced by conditioned medium from senescent cells.^13^ TETRAMER (Supplementary Fig. 4**)** revealed in this system the induction of an immediate early gene program involving proto-oncogenes (JUN, JUNB, MYC), suggested not only tumor suppressors (EGR,^14^ KLF6^15^), but also broad-spectrum inflammatory regulators, like the ubiquitin-editing enzyme TNFAIP3.^16^ The early program drives the temporal induction of downstream NF-κB-mediated programs known to link inflammation and cancer (REL, RELB, NFKB, NFKB2),^17^ and the cytokine-mediated programs triggered by IRF1, also regarded as tumor suppressor, as is the late induced TP53. The last observed programming phase involves TFs like CEBPG,^18^ known to suppress oncogene-induced senescence and inflammatory gene expression, MXD1, a regulatory component in the MYC-MAX-MAD network and heat shock factors (DNAJC2, HSF2). Thus, the secretome of senescent cells activates a plethora of gene regulators that apparently substitute for the oncogene-mediated activities in the stepwise model of human primary cell transformation.

As final example, we used TETRAMER to reconstitute TF wiring implicated in the trans-differentiation of B-lymphoma cells to primary macrophages by over-expressing *CEBPA*^19^ (Fig. 2a). In fact, GRN reconstruction revealed the downstream TF regulatory cascade initiated by C/EBPα, thus providing a comprehensive view of the master regulators implicated in this process. This TF regulatory wiring was reconstituted by integrating TF–TGs relationships from three GRN databases (CellNet^1^, CAGE^3^, qcChIP-seq^6^; Fig. 2b). Note both the redundancy (CellNet vs. qcChIP-seq, TFs depicted at top right; CellNet vs. CAGE, TFs specified on left) and the unique retrieval of some TFs (e.g., HIF1A, MAFF) from different databases, illustrating the advantage of incorporating multiple sources of TF–TG relationships. Indeed, albeit in the predicted C/EBPα cistrome (Fig. 2c), *FOSL1* was only retrieved from CAGE, *SPI1* only from CellNet, and *JUNB* only from the qcChIP-seq database, independent ChIP-seq assays demonstrated C/EBPα binding proximal to each gene, but beyond the proximity criterion of the qcChIP-seq collection (enhancer regions < 10 kb distance to TSS), thus explaining why *FOSL1* and *SPI1* are not predicted as C/EBPα targets by this collection (Fig. 2d). The final temporally organized TF network (Fig. 2e) comprises 70 TFs and 222 relationships, revealing also activation of the endogenous *CEBPA*, which may support maintenance of the trans-differentiated cell state, as it is the case for pluripotency factors in MEF-iPSC reprogramming (Supplementary Fig. 3).

Given that, in principle, TETRAMER predicts master regulators for any cell fate transition by evaluating their impact on transcription regulation throughout the reconstructed network, we applied this approach to predict master regulators to each hypothetically possible cell fate transition within a collection of more than 3000 transcriptomes from ~300 cell/tissue types representing 14 different anatomical systems in the human body.^2^ We generated GRNs that corresponded to the transitions predicted to interconvert any cell/tissue type into any other, and predicted the TF networks expected to drive these cell fate transitions (Supplementary Note and Supplementary Fig. 7a**)**. Finally, the degree of similarity among all inferred TF networks per cell/tissue types was evaluated (Tanimoto Index; Supplementary Note) and clustered to reveal the common anatomical origin of the compared cell/tissue systems (Fig. 3a). As a validation step we focused on the transition towards M2 macrophages from a variety of different cell types (~300 cell/tissue types as source; Fig. 3b) and ranked the transition-implicated TFs according to their capacity to act as master regulators (“MRI;” Fig. 3c**;** Supplementary Fig. 5a). In this manner, we aimed at identifying a consensus TFs for driving cell fate transition towards macrophage, in despite of the cell type/tissue in use as source, as well as to overwhelm potential technical aspects implicated on their prediction. TETRAMER identified major players in the transitions from naïve B cells or primary skin fibroblasts to M2 macrophages (Supplementary Fig. 5b), presenting overlapping but non-identical sets of 11 to 19 master regulators—six of which are common to all three transitions—depending on whether the transition to M2 macrophages was initiated from B cells, fibroblasts, or ES cells (Fig. 3d). Comparing the predictions of TETRAMER with previous efforts to define master regulators (Mogrify,^20^ CellNet^1^, D’Alessio et al.^2^ Supplementary Fig. 6a), revealed a core of TFs commonly predicted in most approaches (Fig. 3d**;** Supplementary Fig. 6b), top ranked on the consensus strategy assessed over ~300 cell/tissue types (Fig. 3c). Of note, CEBPA was identified by all but one approach, clearly suggesting that even the most evident master regulators can also be missed under defined algorithmic/ cell/tissue types, thus supporting the consensus strategy.

**Fig. 3:**
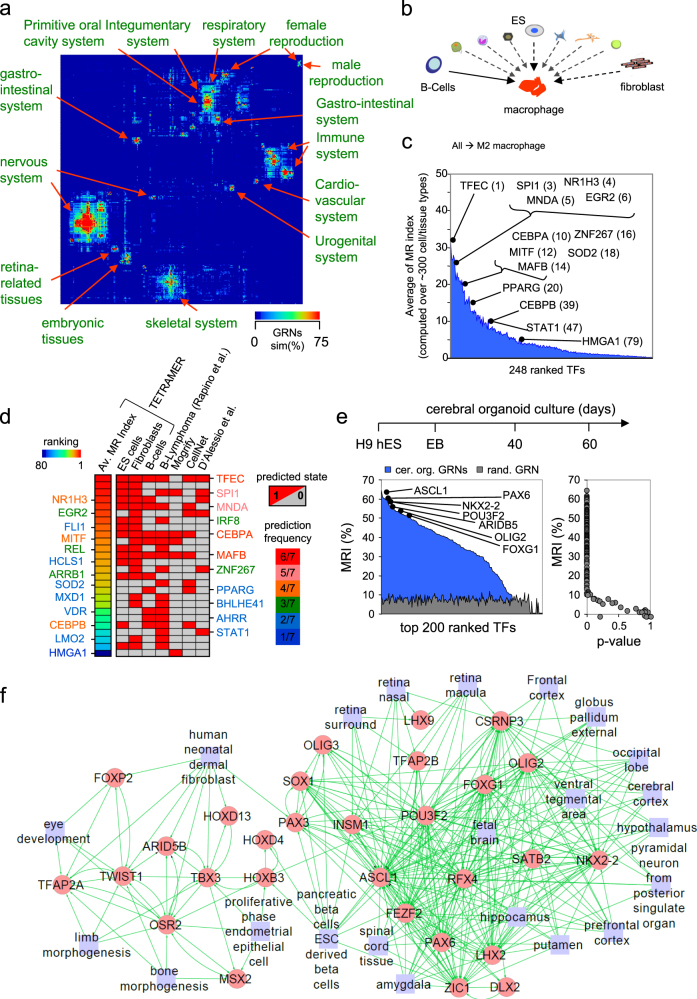
TETRAMER reveals the differential TF networks in hundreds of cell types, predicts candidates of master regulators required for cell fate conversions and identifies TF networks operative during organoid differentiation. **a** TF GRN similarity matrix computed from transcriptomes of ~300 cell/tissue types representing 14 different anatomical systems in the human body. TETRAMER-predicted master regulator GRNs were clustered by their similarity (Tanimoto Index; “GRNs sim(%);” Average Dot Product distance metrics). **b** Using the predicted master regulators over this large number of cell/tissue types TETRAMER can predict the GRN and TF changes needed for the interconversion between any two cell types, as schematically depicted for the (trans-) differentiation to macrophages. The solid arrow reveals the trans-differentiation that has been experimentally confirmed.^19^ **c** TETRAMER-based ranking of TFs according to their master regulator capacity (MR index) to trans-differentiate any of the cell types into M2 macrophages. **d** TETRAMER-predicted master regulators for trans-differentiation of ES, fibroblast or B cells to macrophages (ranked by MRIs, rainbow heatmap) were compared with the one inferred from the temporal transcriptomes^19^ and those predicted by other methods.^1–3^ Color-coded names of TFs reveal common predictions (“prediction frequency”) by different methods. **e, f** Master regulators predicted from transcriptomes assessed during 60 days of H9 hES-derived cerebral organoid cultures (EB, embryoid bodies). In (**f**), the relevance of major TFs predicted in (**e**) is highlighted through their association to cells/tissues (blue boxes). The identification of a few TFs associated with non-neuroectodermal tissues reveals the previously noted presence of undesired cell fate processes in cerebral organoid cultures.^23^

To assess the versatility of TETRAMER we used it to monitor cell fate transitions in the context of developing cerebral organoids.^21^ Specifically, we used temporal transcriptome data generated from brain organoid cultures during 60 days.^22^ TETRAMER predicted 134 TFs with MRI > 25% (*p* < 10^−7^) (Fig. 3e). Considering the complexity of organoids, we expected that the inferred master regulators would correspond to distinct cell types generated during this process. Indeed, by comparing the list of top ranked master regulators with those retrieved on the reconstructed GRNs associated to the ~300 cell/tissue types discussed above (Fig. 3a and Supplementary Fig. 7) several predicted TFs were associated with GRNs operative in a variety of neuronal cell types albeit some were also associated with non-neuroectodermal cell types (Fig. 3f). This last aspect was also observed in immunofluorescence assays with cultured cerebral organoids.^23^ Taken altogether, this effort illustrates the potential of TETRAMER to study cell fate transitions even in complex heterogeneous systems, such as developing organs.

While in the last years approaches for predicting master regulators, as molecular targets of pharmacologically relevant compounds, were developed on the basis of static or dynamic inferred gene-regulatory maps (DeMAND,^24^ PROTINA^25^), their accessibility by the scientific community is restricted by the requirements of using numerical computing environments (including in some cases the necessity of having a commercial license). In contrast, TETRAMER is freely available through the Cytoscape App Store (http://apps.cytoscape.org/apps/tetramer), and from a dedicated website providing access to the various networks described in this study (http://igbmc.fr/Gronemeyer/qcgenomics/TETRAMER). Through this platform, users have the possibility to query predicted MRs for (i) a given cell transformation; (ii) the transformation of a given cell towards any other cell type; and (iii) the transformation of a given cell into any other cell. Moreover, users can compare the outputs generated by the Cystoscape app with the collection of GRNs reconstructed for ~300 human cell/tissue types.

## Electronic supplementary material

Supplementary Figures

Supplementary notes

## Electronic supplementary material

**Supplementary information** accompanies the paper on the *npj Systems Biology and Applications* website (10.1038/s41540-018-0066-z).
