# Supplementary material for: Modeling gene-regulatory networks to describe cell fate transitions and predict master regulators
Source: NPJ Syst Biol Appl. 2018 Aug 2;4:29. doi: 10.1038/s41540-018-0066-z (PMC6070484; doi:10.1038/s41540-018-0066-z)
Supplement: Supplementary file 1 — Supplementary Figures [file 41540_2018_66_MOESM1_ESM.pdf]

Modeling gene-regulatory networks to describe cell fate transitions and predict master regulators

Pierre-Etienne Cholley[1], Julien Moehlin, Alexia Rohmer, Vincent Zilliox, Samuel Nicaise, Hinrich Gronemeyer* and Marco Antonio Mendoza-Parra [2,*]

Equipe Labellisée Ligue Contre le Cancer, Department of Functional Genomics and Cancer, Institut de Génétique et de Biologie Moléculaire et Cellulaire (IGBMC), Centre National de la Recherche Scientifique UMR 7104, Institut National de la Santé et de la Recherche Médicale U964, University of Strasbourg, Illkirch, France.

[1] Current affiliation: Computational Systems Biology Infrastructure, Chalmers University of Technology, Kemivägen 10, 41296, Gothenburg, Sweden.

[2] Current affiliation: UMR 8030 Génomique Métabolique, Genoscope, Institut François Jacob, CEA, CNRS, University of Evry-val-d'Essonne, University Paris-Saclay, 91057 Évry, France.

*Corresponding authors:
Marco Antonio Mendoza-Parra
E-mail: marco@igbmc.fr
Hinrich Gronemeyer
E-mail: hg@igbmc.u-strasbg.fr
Phone: +(33) 3 88 65 34 73
Fax: +(33) 3 88 65 34 37

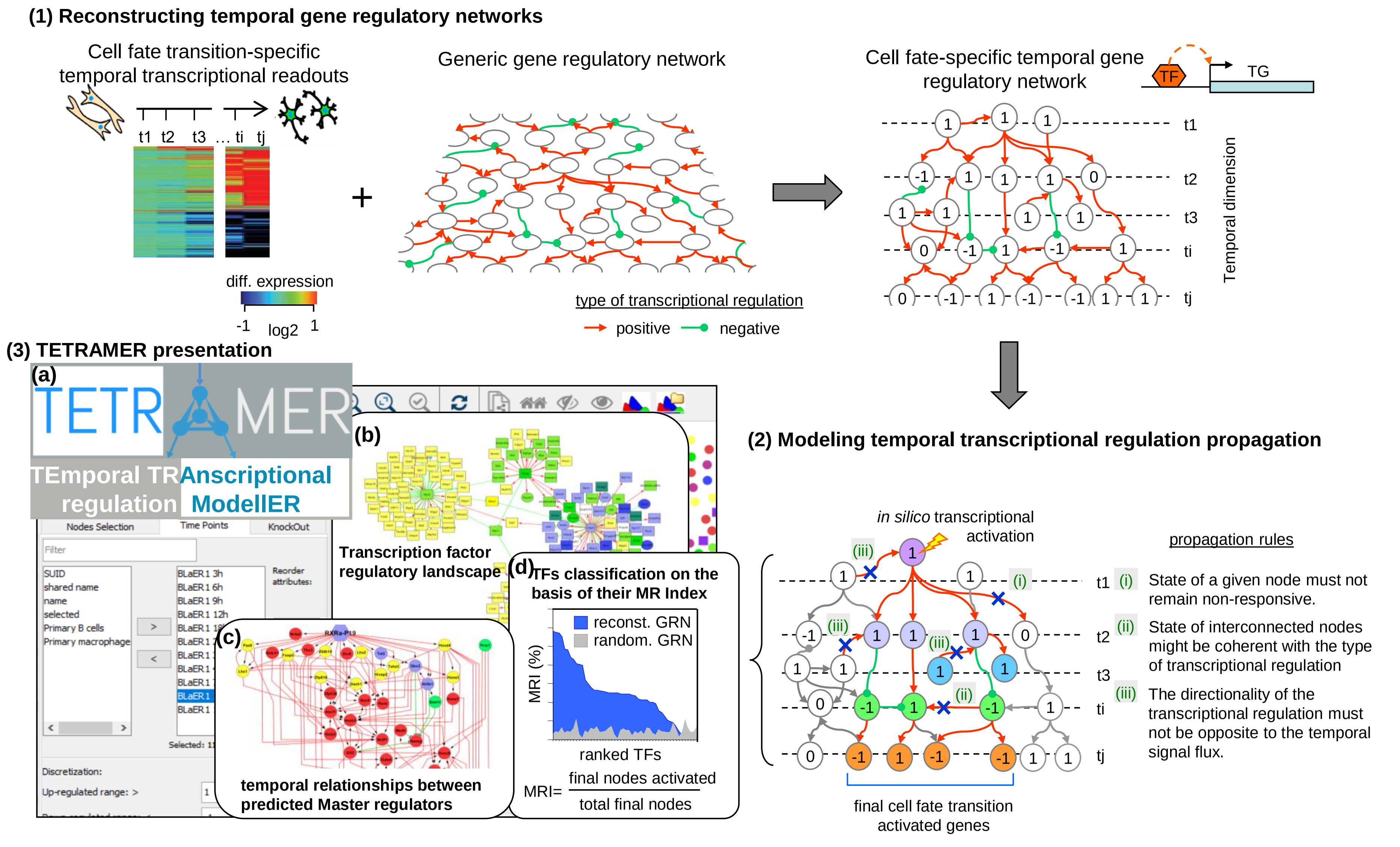

Supplementary Figure 1. The TETRAMER workflow. (1) Temporal transcriptome readouts are integrated with generic gene regulatory networks (GRN) to reconstruct a cell fate-specific temporal GRN. **(2)** Over the reconstructed topology, each transcription factor (TF) is evaluated for its capacity to drive a cascade of temporal transcription regulation events towards a subset of, or all genes that specify, for instance, the final state of the cell fate transition. Such *in silico* propagation follows three logical 'propagation' rules (i to iii, right panel), as illustrated in the network sketch [x, 'forbidden' edges according to rules I, ii or iii; colored nodes depict the flux of transcription regulatory information from the initial cue (flash) towards the final state at time point 'tj']. On the basis of such propagation results, a Master Regulator Index (MRI) is computed for each TF, which is then used for ranking (depicted in 3d). **(3)** TETRAMER is available using the Cytoscape environment: **(a)** illustration of the TETRAMER control panel in cytoscape; **(b)** display of a regulatory landscape of a ranked TF produced by TETRAMER; **(c)** Temporal transcription regulatory relationships among multiple TFs; **(d)** which is generated from the ranking on the basis of their MRIs predicted by TETRAMER.

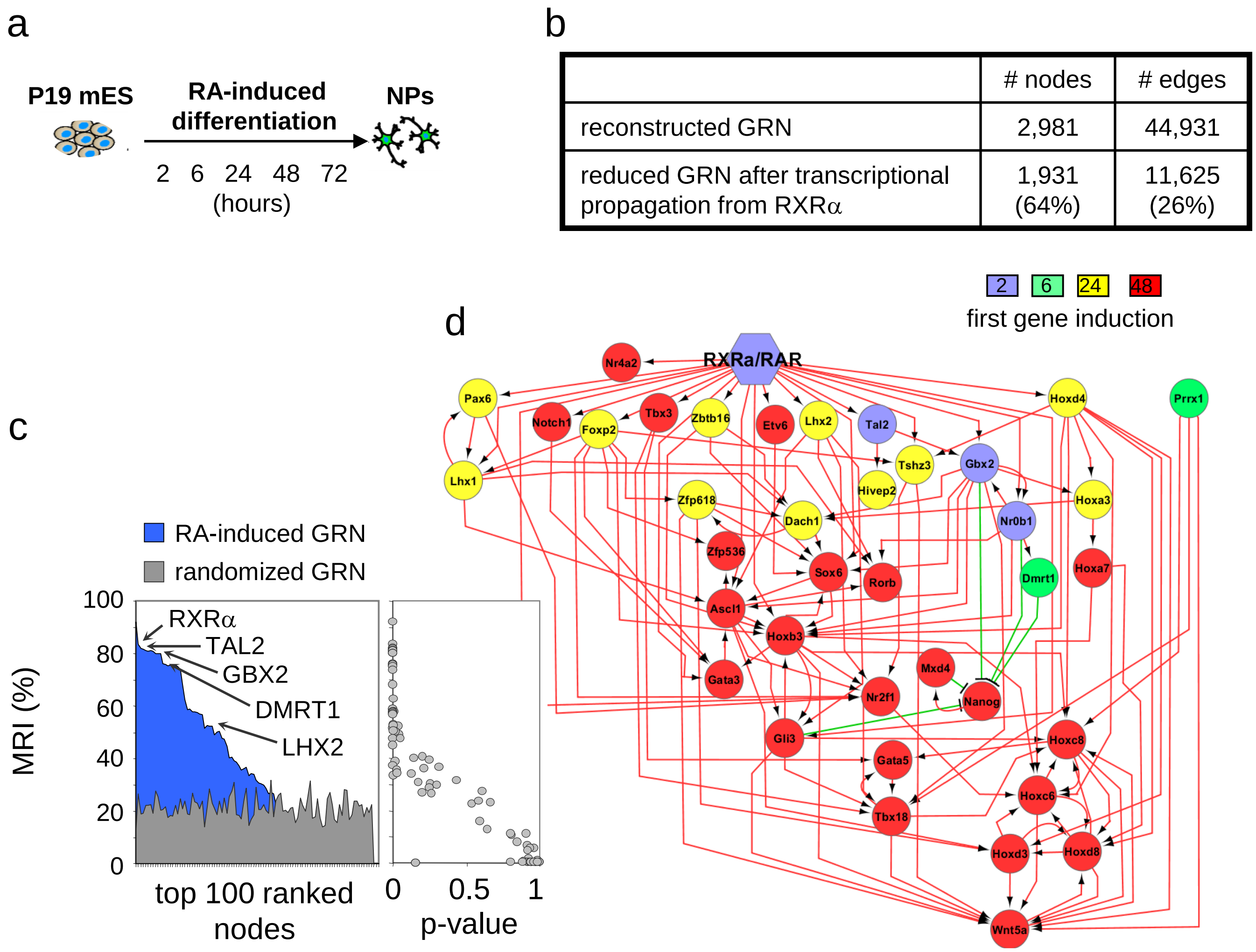

Supplementary Figure 2. Master regulator network driving neuronal cell fate acquisition.
(a) Temporal transcriptome changes during neuronal precursor (NPs) cell differentiation induced by retinoic acid (RA) treatment of P19 cells has been used for reconstructing a gene-regulatory network (GRN)[1]. **(b)** A temporal transcriptional signaling propagation initiated *in silico* with RAR/RXRα gave rise to a reduced GRN of that has been used for predicting major master regulators by raking the TFs on the basis of their Master Regulator Index (MRI). **(c)** Ranking the top100 TFs on the basis of their MRIs identified a subset of 39 nodes with MRIs > 40% (P-value < $1x10^{-10}$). Multiple randomizations of the network connectivities supported the significance of the predicted MRIs (grey shaded) . Among the top ranked nodes, in addition to well-known neurogenic players, TFs like TAL2, GBX2, DMRT1 or LHX2 were predicted as master regulators. **(d)** This master regulator network, generated by TETRAMER, displays the temporal induction of each of the predicted TFs, illustrating their temporal transcription regulatory relationships. Color code, time of first gene induction, as depicted at the top.

a

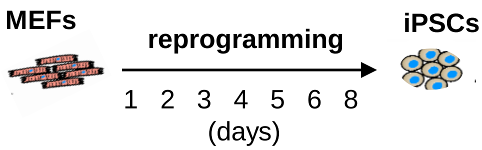

b

Days	D1	D2	D3	D4	D5	D6	D8
Diff. Genes	14	46	38	31	51	200	1129

	# nodes	# edges
reconstructed GRN	20,702	558,130

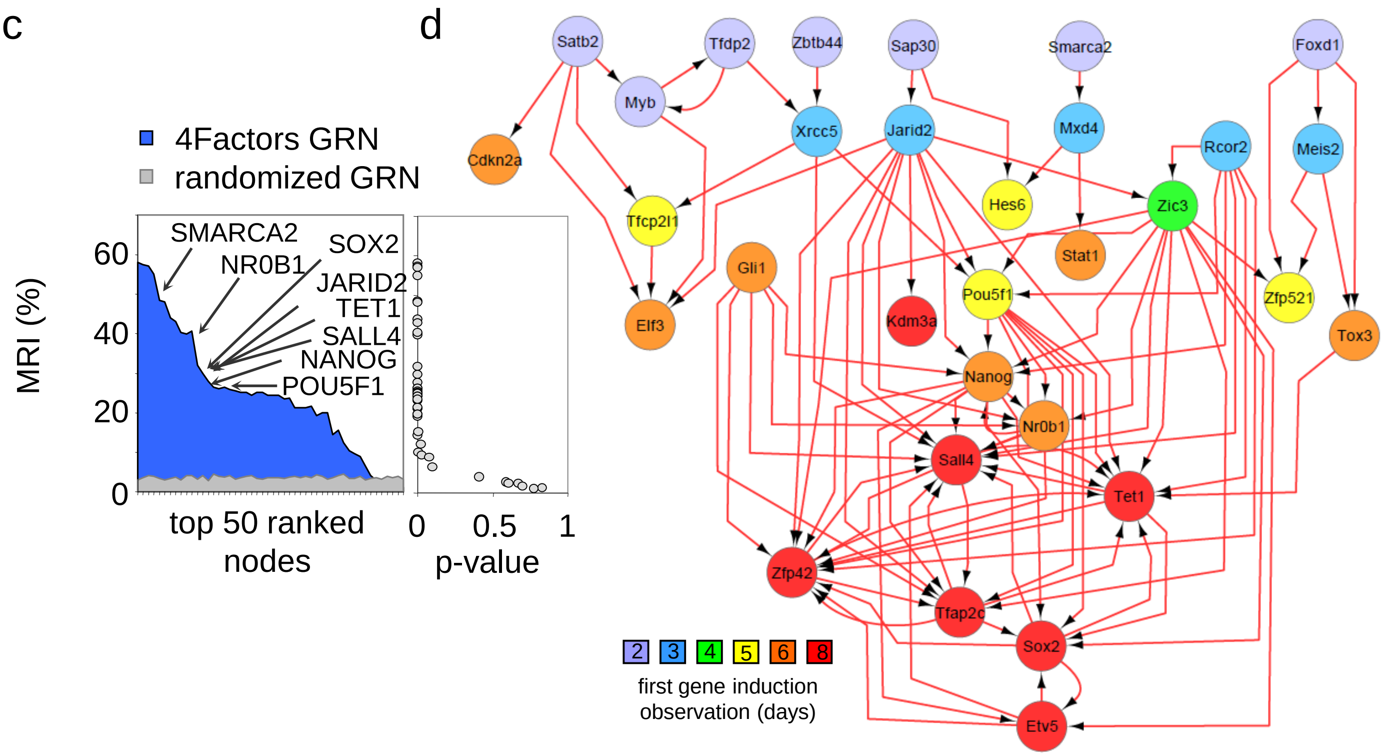

Supplementary Figure 3. Master regulatory network driving reprogramming towards induced pluripotent stem cells. (a) Re-analysis of the temporal transcriptomes assessed by Koga and colleagues during reprogramming of mouse embryonic fibroblast to iPSCs by the ectopic over-expression of *Oct4, Sox2, Klf4* and *c-Myc*[2]. **(b)** TETRAMER reconstructed a GRN composed of 20,702 nodes and 558,130 edges from 7 temporal transcriptomes during the first 8 days of re-programming. **(c)** Monitoring *in silico* the impact of TF on the temporal evolution of transcriptomes from MEFs to iPSCs by TETRAMER identified 21 TFs with MRIs > 25% (p-value <1x10^{-10}). **(d)** TETRAMER-generated master regulator network reveals the evolution of cross-regulation between predicted TFs. Note that expression of the endogenous self-renewal factors SALL4, SOX2, NANOG, NR0B1 or POU5F1/OCT4 is observed from the fifth day on. Furthermore, a number of early responding factors, like FOXD1, the chromatin remodeling components SAP30[3], SATB2[4] or SMARCA2 are also retrieved from this network, suggesting reorganization of the epigenetic landscape during reprogramming.

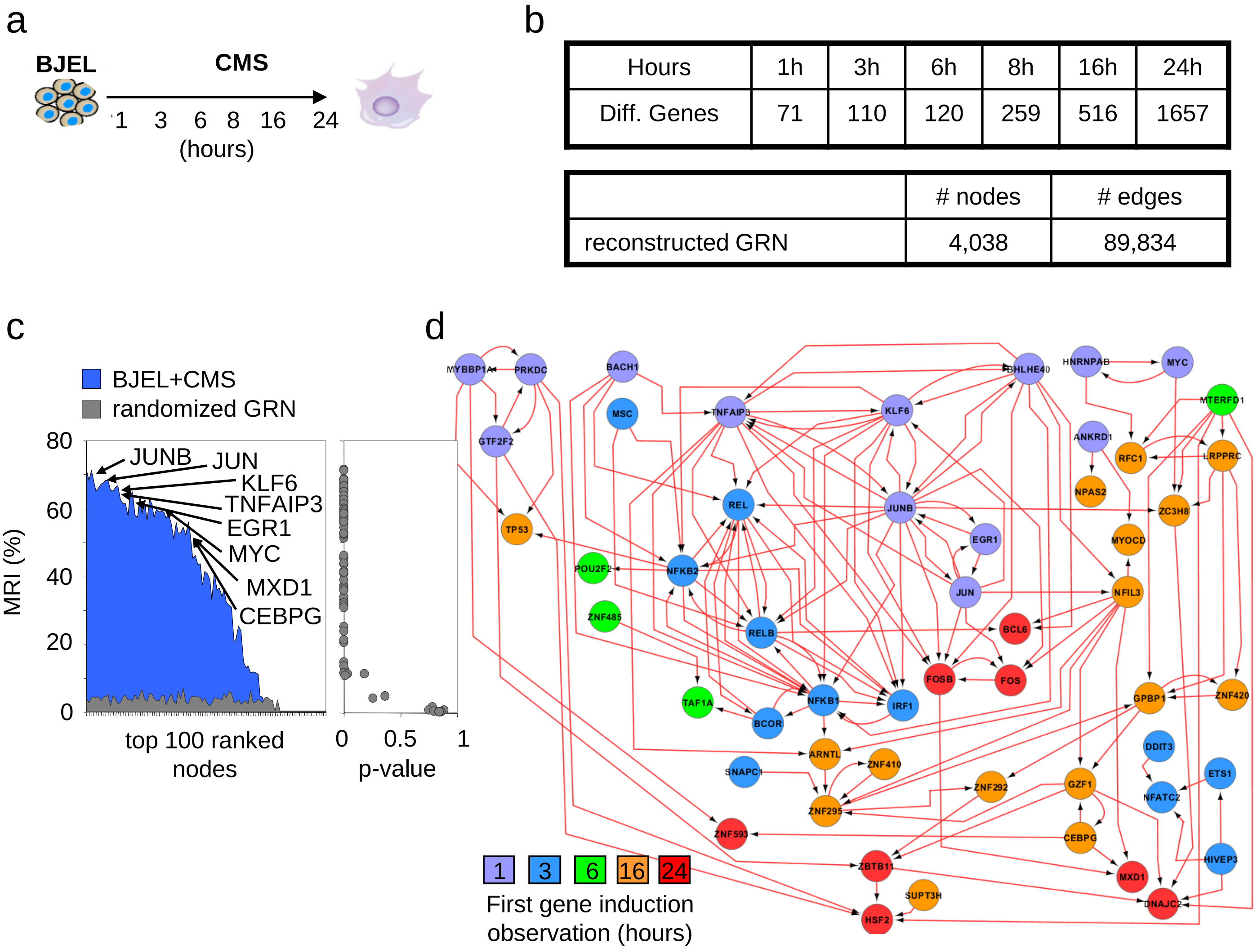

Supplementary Figure 4. Transcription factors involved in the tumorigenic transformation of pre-transformed fibroblast cells by conditioned medium from senescent cells. (a) Re-analysis of the temporal transcriptomes assessed by Vjetrovic and colleagues during tumorigenic transformation of immortalized human foreskin BJ fibroblasts (BJEL) by conditioned medium from senescent cells (CMS)[5]. **(b)** Temporal transcriptomes covering the first 24h of exposure to CMS were used for reconstructing a GRN composed of ~4,000 nodes and >89,000 edges. **(c)** The *in-silico* temporal transcription propagation assay by TETRAMER identified 53 TFs with MRIs > 10% (p-value $<1X10^{-50}$). **(d)** A reconstructed gene regulatory network of major TFs reveals early activation of a program involving the proto-oncogenes JUN, JUNB and MYC, as well as inflammatory regulators like TNFAIP3. At later stages, factors like C/EBPγ, implicated on the suppression of oncogene-induced senescence or MXD1, a component of the MYC-MAX-MAD network, were observed.

a

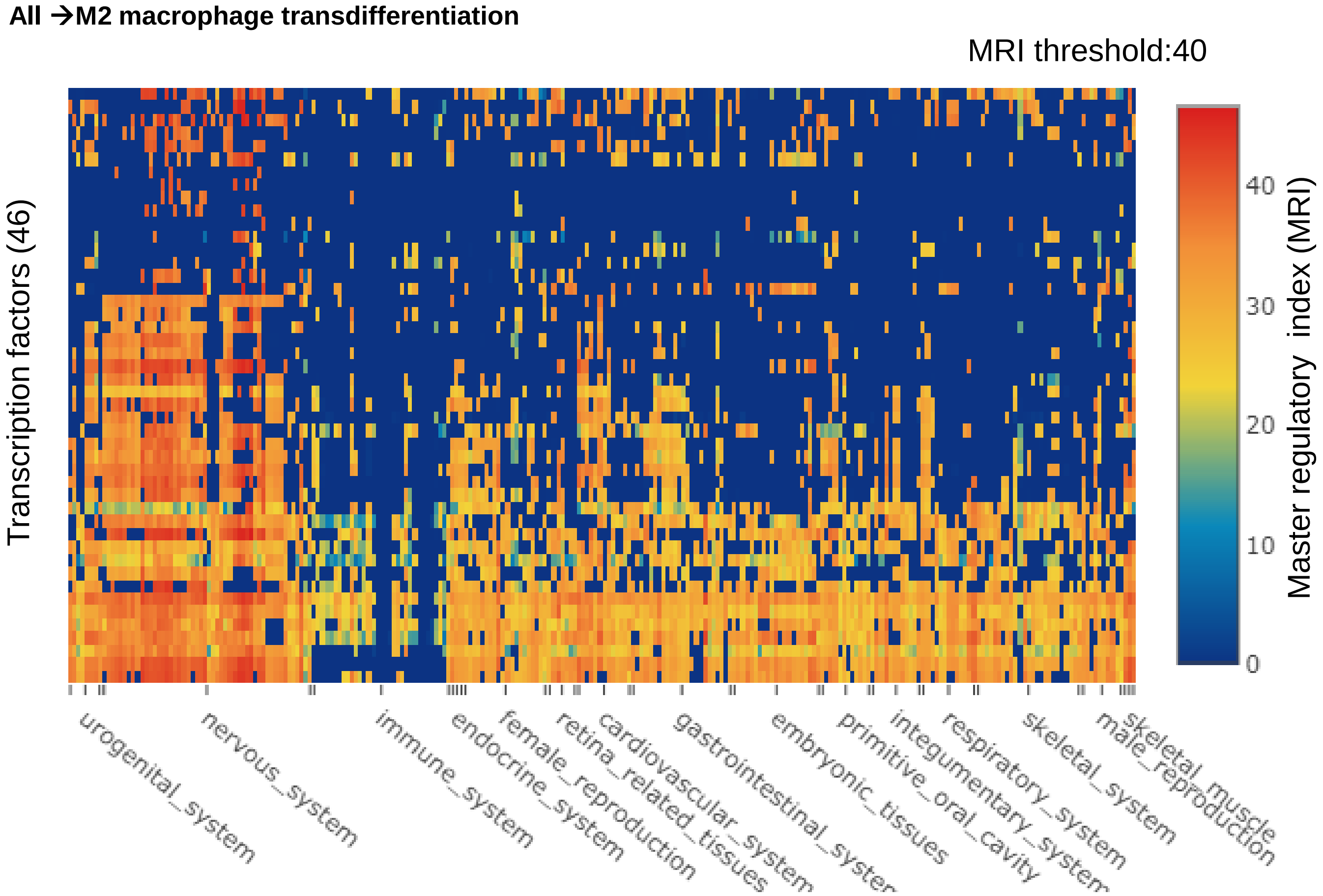

b

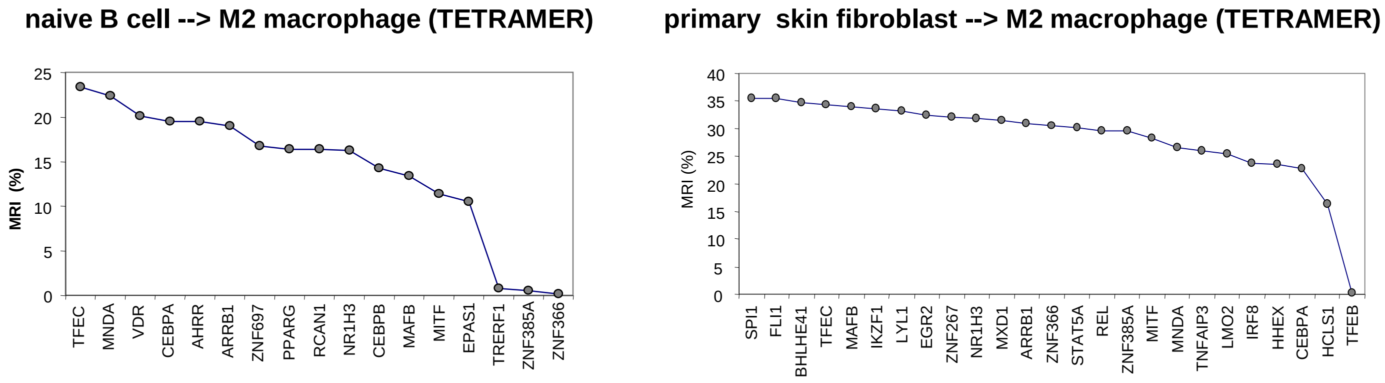

Supplementary Figure 5. Predicted Master regulators implicated in trans-differentiation of diverse cells to macrophages. (a) Heat-map displaying a set of 46 TFs presenting master regulator characteristics as predicted from an ensemble of ~300 cell types (MRI threshold: 40; i.e. with at least an MRI per cell/tissue type higher than 40%). **(b)** Master Regulatory Index (MRI) predicted by TETRAMER for various TFs suggesting their capacity to 'drive' naive B cells or primary skin fibroblasts towards M2 macrophages. All presented data is available from the dedicated website (http://igbmc.fr/Gronemeyer/qcgenomics/TETRAMER).

a

**Methods**	**TF-TG source**	**Approach**	**Systems available**	**User's data analysis**	**Temporal analysis**	**Platform accessibility**
**CellNet[7]**	Differential Gene expression (microarrays)	Gene regulatory networks	Mouse cell/tissue types (20); human cell /tissue types (16)	Yes	No	Dedicated website
**D'allesio[8]**	Differential Gene expression (microarrays)	TF-expression levels classification	Human cell/tissue types (~300)	No	No	None
**Mogrify[6]**	CAGE RNA-seq + STRING database; DNA binding Motif analysis	Gene regulatory networks	Human cell/tissue types (>300)	No	No	Dedicated website
**TETRAMER**	ChIP-seq, microarrays; CAGE	Gene regulatory networks	Human; mouse; user-defined	Yes	Yes	Cytoscape App & Dedicated website

b

Source Cell Type: Bcell **Target Cell Type:** Macrophage

**Mogrify**	**CellNet**	**D-Alessio et al**
MITF	**MAFB**	**TFEC**
**SPI1**	CEBPB	STAT1
**CEBPA**	MNDA	EGR2
**MAFB**	**CEBPA**	IRF1
DBP	TFEC	ASCL2
ETS2	EGR2	**SPI1**
SNAI3	PPARG	ZNF267
HMGA1	SOD2	NR1H3

Supplementary Figure 6. (a) Comparison between the various methodologies described for predicting master regulatory TFs. **(b)** Table summarizing the major TFs predicted by Mogrify, CellNet or the study performed by d'Alessio et al (source: Supplementary file; Mogrify[6]).

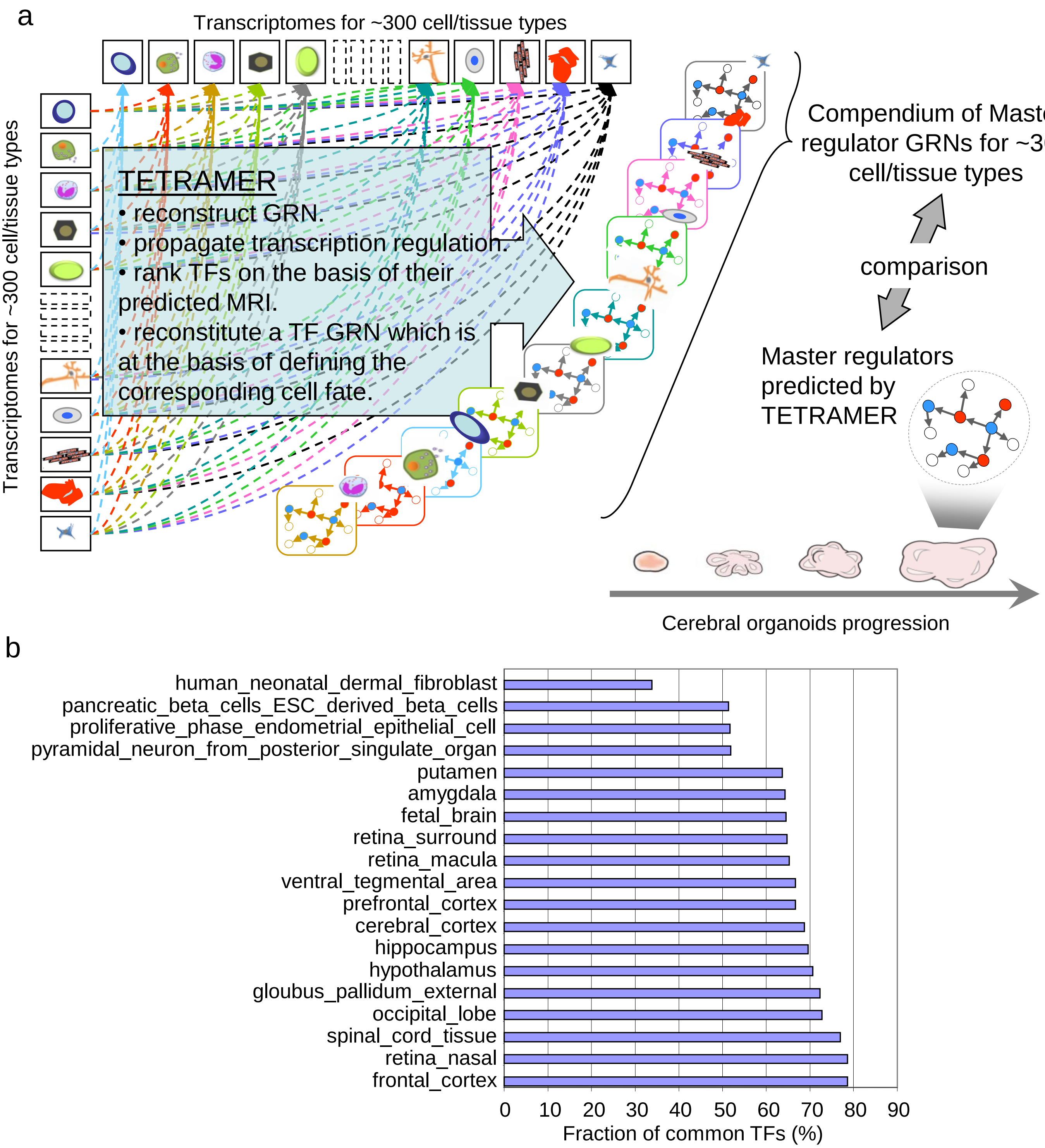

Supplementary Figure 7. Relevance of the predicted Master regulators during cerebral organoid progression in the context of GRNs established for ~300 cell systems.
(a) TETRAMER predicted Master regulator TFs driving cell fate conversions among a collection of ~300 cell/tissue types. As consequence, a compendium of master regulator GRNs has been established which can be used for comparative purposes with any other cell fate transition study. **(b)** Master regulators predicted from transcriptomes assessed during 60 days of H9 hES-derived cerebral organoid cultures[9] (MRI > 25%) were compared with the compendium of Master regulator GRNs for revealing the presence of heterogeneous cell systems on the developed organoid. The illustrated bar plot represents the fraction of master regulators in common with the queried cell/tissue system relative to the total number of TFs per system.

REFERENCES

1. Mendoza-Parra, M.A. et al. Reconstructed cell fate-regulatory programs in stem cells reveal hierarchies and key factors of neurogenesis. *Genome Res 26, 1505-19* (2016).

2. Koga, M. et al. Foxd1 is a mediator and indicator of the cell reprogramming process. Nat Commun 5, 3197 (2014).

3. Li, Dongwei et al. Chromatin Accessibility Dynamics during iPSC Reprogramming. Cell Stem Cell, Volume 21, 6, 819-833.e6 (2017).

4. Zhou P, Zhou P, Wu G, Zhang P, et al. SATB2-Nanog axis links age-related intrinsic changes of mesenchymal stem cells from craniofacial bone. *Aging (Albany NY)*; 8(9) (2016).

5. Vjetrovic, J., Shankaranarayanan, P., Mendoza-Parra, M.A. & Gronemeyer, H. Senescence-secreted factors activate Myc and sensitize pretransformed cells to TRAIL-induced apoptosis. *Aging Cell 13(3), 487-96* (2014).

6. Rackham, O., et al. A predictive computational framework for direct reprogramming between human cell types. Nature Genetics 48, 331-335 (2016).

7. Cahan, P. et al. CellNet: network biology applied to stem cell engineering. *Cell* 158, 903-915 (2014).

8. D'Alessio, A.C. et al. A Systematic Approach to Identify Candidate Transcription Factors that Control Cell Identity. *Stem Cell Reports* 5, 763-775 (2015).

9. Luo, C. et al. Cerebral Organoids Recapitulate Epigenomic Signatures of the Human Fetal Brain. *Cell Rep* 17, 3369-3384 (2016).
