# Supplementary material for: Modeling gene-regulatory networks to describe cell fate transitions and predict master regulators
Source: NPJ Syst Biol Appl. 2018 Aug 2;4:29. doi: 10.1038/s41540-018-0066-z (PMC6070484; doi:10.1038/s41540-018-0066-z)
Supplement: Supplementary file 2 — Supplementary notes [file 41540_2018_66_MOESM2_ESM.doc]

**Supplementary Note**

**Modeling gene-regulatory networks to describe cell fate transitions and predict master regulators**

**Pierre-Etienne Cholley^1^, Julien Moehlin, Alexia Rohmer, Vincent Zilliox,**

**Samuel Nicaise, Hinrich Gronemeyer* and Marco Antonio Mendoza- Parra^2^,***

Equipe Labellisée Ligue Contre le Cancer, Department of Functional Genomics and Cancer, Institut de Génétique et de Biologie Moléculaire et Cellulaire (IGBMC), Centre National de la Recherche Scientifique UMR 7104, Institut National de la Santé et de la Recherche Médicale U964, University of Strasbourg, Illkirch, France.

1 Current affiliation: Computational Systems Biology Infrastructure, Chalmers University of Technology, Kemivägen 10, 41296, Gothenburg, Sweden.

2 Current affiliation: UMR 8030 Génomique Métabolique, Genoscope, Institut François Jacob, CEA, CNRS, University of Evry-val-d’Essonne, University Paris-Saclay, 91057 Évry, France.

*Corresponding authors:

Marco Antonio Mendoza-Parra

E-mail: [marco@igbmc.fr](mailto:marco@igbmc.fr)

Hinrich Gronemeyer

E-mail: [hg@igbmc.u-strasbg.fr](mailto:hg@igbmc.u-strasbg.fr)

Phone: +(33) 3 88 65 34 73

Fax: +(33) 3 88 65 34 37

**1.0. TETRAMER Workflow**

TETRAMER has been implemented in a Cytoscape App providing an optimal user-friendly framework for (i) the reconstruction of cell fate transition-specific Gene regulatory networks (GRNs) and (ii) the prediction of relevant TFs by evaluating their capacity to drive a cascade of temporal transcription regulatory events implicating the largest number of downstream TGs.

**1.1. Building GRNs with TETRAMER**

TETRAMER builds a GRN from the temporal transcription information (transcriptomes) provided by the user. This is done by integrating the list of differentially expressed genes assessed from temporal transcriptomes with a collection of generic TF-TGs relationships collected from the public domain. Specifically, TETRAMER requires a text file containing the temporal differential expression levels (fold change in log2; as described in the corresponding [Tutorial](http://archive.igbmc.fr/recherche/Prog_FGC/Eq_HGron/Bioinfotools/Tetramer/Tutorial.php)). It is worth to mention that the current version of TETRAMER does not handle replicates, thus users are invited to provide an averaged fold-change value per replicates. This last aspect might be part of the future upgrades such that the data variance could be considered within network reconstruction but also over transcription signal propagation.

Currently, TETRAMER allows to reconstruct GRNs by integrating the information obtained from three TF-TG connectivity sources:

1. ***CellNET:*** TF-TG relationships assembled by *Cahan et al* from the analysis of ~100 mouse or ~60 human microarray transcriptomes covering 20 mouse or 16 human cell/tissue types respectively. Both mouse and human collection are available in TETRAMER^1^.
2. ***qcGenomics:*** TF-TG relationships established from publicly available ChIP-sequencing studies and quality-assessed using the [NGS-QC](http://www.ngs-qc.org/) Generator^2^. Currently 2332 mouse or 3913 human TF ChIP-sequencing datasets were integrated in TETRAMER for making a mouse and human collection. These generic GRNs are constructed by associating the most confident TF binding sites (MACS2 peak calling; p-value threshold: 1x10^-50^) to their closest promoters (2.5, 10 or 20kb, such that users might have the possibility to interrogate for promoter regions or proximal promoter enhancer regions if wished). Furthermore, a minimal quality score of CCC (quality range from A to D; see [NGS-QC](http://www.ngs-qc.org/) Generator^2^) is required for all ChIP-seq profiles that are included in this analysis.
3. ***regulatoryCircuits:*** TF-TG relationships established by Marbach D. et al from the cap analysis of gene expression (CAGE) performed by the FANTOM consortium combined with sequencing binding motif analysis^3^. This resource comprises GRNs for 394 human cell types/tissues, which have been combined in a single GRN.

Users have the possibility to include their own TF-TG interaction file for custom applications. The procedure to follow is described on the tutorial available in the dedicated website for TETRAMER.

#### 1.2. Modeling temporal transcription regulatory signaling propagation

Once a GRN is reconstructed, TETRAMER models a temporal transcription regulation cascade from a set of defined "start nodes" towards the "final nodes", which correspond to the transcriptome when the cell fate transition has been completed (e.g. cell differentiation, reprogramming; etc). This signal propagation takes place through the defined network structure and in coherence with the differential expression state of each node at each defined time point. Furthermore, the temporal transcriptional response has to be coherent with the type of transcription regulation (positive or negative; i.e. transcription activation or repression) corresponding to the assessed TF-TG relations. In brief, TETRAMER evaluates the capacity of each factor that has been defined as "start node" to propagate the transcription response within the network to the maximal number of "final nodes"; this capacity is expressed as “Master Regulator Index (MRI)”, which is defined as the fraction (%) of final nodes regulated by a given TF relative to the total number of final nodes. The MRI is complemented by a second metrics, the Master Regulator Specificity (MRS), which corresponds to the fraction of nodes regulated by a given TF relative to the total number of nodes at a defined time-point. The MRS evaluates whether a TF could in fact regulate other nodes beyond those in the "final list", thus providing a further level of complexity for the characterization of the TF regulatory influence within the reconstructed network. Finally, TFs can ranked either on the basis of the product of the MRI and MRS, or on the basis of MRI alone .

$$MRI=\left(\frac{regulated\_nodes\cap final\_nodes}{final\_nodes}\right)x100$$

$$MRS=\left(\frac{regulated\_nodes\cap final\_nodes}{regulated\_nodes}\right)x100$$

**1.3. Evaluating the confidence of the predicted Master regulators**

Given that TETRAMER incorporates transcription regulatory relationships obtained from the public domain, the likelihood of incorporating non-relevant edges during the network reconstruction needs to be evaluated. Modeling transcription propagation addresses this issue, as only edges coherent with the transcriptional state of the associated node - in the corresponding temporal context - are kept. To further support the statistical significance of this procedure, TETRAMER randomizes all edge associations within the entire network and re-evaluates subsequently MRI and MRS data for all retrieved factors in the context of the randomized network. A large number of randomizations are used (10 to 100 times) to obtain averaged randomized MRI and MRS data and attribute confidence values to the master regulator descriptors (MRI and MRS) relative to the randomized distribution (Z score statistical test).

#### 2.0 Installation and usage

TETRAMER has been designed for Cytoscape version 3.4 and higher. The corresponding JAR file is available for download [in](http://archive.igbmc.fr/recherche/Prog_FGC/Eq_HGron/Bioinfotools/Tetramer/Download.php) the dedicated TETRAMER website, as well as from the Cytoscape app repository <http://apps.cytoscape.org/apps/tetramer>. To have a detailed explanation concerning the installation and use of TETRAMER, please refer to the corresponding tutorial at <http://igbmc.fr/Gronemeyer/qcgenomics/TETRAMER>.

#### 3.0. Application cases

***3.1 Predicting Master regulator co-regulatory networks in various cell fate transition events***

The concept behind TETRAMER has been initially designed for the study of retinoid-driven neuronal/endodermal cell fate decisions^4^. In this study, the propagation of the temporal transcription regulation events has been modeled (customized R script released in^4^) over a GRN reconstructed by the integration of the collected temporal transcriptome readouts (GSE68291) and the generic TF-TG relationships retrieved in CellNet^1^. This initial effort, has been generalized by the establishment of the current Cytoscape App and applied to studies concerning OSKM-driven cell reprogramming^5^ (GSE38509), trans-differentiation^6^ (GSE44700), aberrant tumorigenic transformation^7^ (GSE49944), as well as complex human brain organoid cultures^8^ (GSE82022).

***3.2. An atlas of candidate master regulators driving the inter-conversion of ~300 human cell/tissue lines***

In this study, TETRAMER has being used to generate an atlas of candidate master regulators for the inter-conversion, e.g. by reprogramming approaches or trans-differentiation, of more than 300 human cell lines^9^. For it, 3821 CEL files were downloaded from GEO(GSE7307) and normalized altogether with pyaffy (python implementation of the RMA algorithm; <https://github.com/flo-compbio/pyaffy>). Considering that multiple CEL files are associated to a single cell/tissue type, we have computed a mean normalized expression value and its corresponding standard deviation per cell/tissue type under study. Finally a differential expression value among cell/tissue type combinations has been computed and used as input for TETRAMER (batch version of the Cytoscape App). As consequence, 82656 files containing predicted master regulators with their associated MRI and confidence p-value -inferred by GRN edges randomisation (100 iterations)- are produced.

With the aim of enhancing the specificity of the predicted master regulator TFs per cell/tissue type inter-conversion, we have evaluated the frequency of over-expression of TFs over all studied cell/tissue types. This frequency criterion (normalized relative to the total number of cell/tissue types) as well as the inferred confidence is used to correct the predicted MRIs as following:

$$wMRI=MRI*(1-freq)*(1-pvalue)$$

where wMRI correspond to the "weighted MRI". As consequence, the wMRI describes the capacity of a given TF to drive the cell/tissue type inter-conversion in an especific context, i.e. relative to all other considered cell/tissue type inter-conversion events.

This analytical procedure allows to identify set of MR TFs specific for each cell/tissue type inter-conversion. Importantly, cell/tissue types can also be considered as part of functionally/anatomically related systems, an aspect that can be reconstituted by a similarity analysis among the predicted master regulators. For it, we have computed pair-wise Tanimoto Similarity index among all predicted sets of MR TFs per cell/tissue types. Specifically this is performed by the following metrics:

$$Tindex=\frac{MRa\cap MRb}{MRa+MRb-(MRa\cap MRb)}$$

where MRa or MRb correspond to the number of Master regulators predicted for the conversion towards the cell/tissue type a or b.

#### 4.0. References

1. Cahan, P. et al. CellNet: network biology applied to stem cell engineering. *Cell* **158**, 903-915 (2014).

2. Mendoza-Parra, M.A., Van Gool, W., Mohamed Saleem, M.A., Ceschin, D.G. & Gronemeyer, H. A quality control system for profiles obtained by ChIP sequencing. *Nucleic Acids Res* **41**, e196 (2013).

3. Marbach, D. et al. Tissue-specific regulatory circuits reveal variable modular perturbations across complex diseases. *Nat Methods* **13**, 366-370 (2016).

4. Mendoza-Parra, M.A. et al. Reconstructed cell fate-regulatory programs in stem cells reveal hierarchies and key factors of neurogenesis. *Genome Res* ***26****, 1505-19* (2016).

5. Koga, M. et al. Foxd1 is a mediator and indicator of the cell reprogramming process. *Nat Commun* **5**, 3197 (2014).

6. Rapino, F. et al. C/EBPalpha induces highly efficient macrophage transdifferentiation of B lymphoma and leukemia cell lines and impairs their tumorigenicity. *Cell Rep* **3**, 1153-1163 (2013).

7. Vjetrovic, J., Shankaranarayanan, P., Mendoza-Parra, M.A. & Gronemeyer, H. Senescence-secreted factors activate Myc and sensitize pretransformed cells to TRAIL-induced apoptosis. *Aging Cell* ***13****(3), 487-96* (2014).

8. Luo, C. et al. Cerebral Organoids Recapitulate Epigenomic Signatures of the Human Fetal Brain. *Cell Rep* **17**, 3369-3384 (2016).

9. D'Alessio, A.C. et al. A Systematic Approach to Identify Candidate Transcription Factors that Control Cell Identity. *Stem Cell Reports* **5**, 763-775 (2015).
